# The influence of social and cultural practices on maternal mortality: a qualitative study from South Punjab, Pakistan

**DOI:** 10.1186/s12978-021-01151-6

**Published:** 2021-05-18

**Authors:** Sonia Omer, Rubeena Zakar, Muhammad Zakria Zakar, Florian Fischer

**Affiliations:** 1 Department of Public Health, Institute of Social and Cultural Studies, University of the Punjab, Lahore, Pakistan; 2 University of Okara, Okara, Pakistan; 3 Institute of Public Health, Charité – Universitätsmedizin Berlin, Berlin, Germany; 4 Institute of Gerontological Health Services and Nursing Research, Ravensburg-Weingarten University of Applied Sciences, Weingarten, Germany

**Keywords:** Maternal health, Reproductive health, Sexual and reproductive rights, Tradition, Culture

## Abstract

**Background:**

A disproportionately high rate of maternal deaths is reported in developing and underdeveloped regions of the world. Much of this is associated with social and cultural factors, which form barriers to women utilizing appropriate maternal healthcare. A huge body of research is available on maternal mortality in developing countries. Nevertheless, there is a lack of literature on the socio-cultural factors leading to maternal mortality within the context of the Three Delays Model. The current study aims to explore socio-cultural factors leading to a delay in seeking care in maternal healthcare in South Punjab, Pakistan.

**Methods:**

We used a qualitative method and performed three types of data collection with different target groups: (1) 60 key informant interviews with gynaecologists, (2) four focus group discussions with Lady Health Workers (LHWs), and (3) ten case studies among family members of deceased mothers. The study was conducted in Dera Ghazi Khan, situated in South Punjab, Pakistan. The data was analysed with the help of thematic analysis.

**Results:**

The study identified that delay in seeking care—and the potentially resulting maternal mortality—is more likely to occur in Pakistan due to certain social and cultural factors. Poor socioeconomic status, limited knowledge about maternal care, and financial constraints among rural people were the main barriers to seeking care. The low status of women and male domination keeps women less empowered. The preference for traditional birth attendants results in maternal deaths. In addition, early marriages and lack of family planning, which are deeply entrenched in cultural values, religion and traditions—e.g., the influence of traditional or spiritual healers—prevented young girls from obtaining maternal healthcare.

**Conclusion:**

The prevalence of high maternal mortality is deeply alarming in Pakistan. The uphill struggle to reduce deaths among pregnant women is firmly rooted in addressing certain socio-cultural practices, which create constraints for women seeking maternal care. The focus on poverty reduction and enhancing decision-making power is essential for supporting women’s right to medical care.

**Supplementary Information:**

The online version contains supplementary material available at 10.1186/s12978-021-01151-6.

## Plain language summary

Round the world, many women are dying because of complications during pregnancy or in childbirth. These deaths are more frequent in developing and underdeveloped countries. Some reasons for this are related to social and cultural factors, which form barriers to women using appropriate maternal healthcare. Therefore, this study aims to explore socio-cultural factors leading to a delay in seeking maternal healthcare in South Punjab, Pakistan.

We interviewed a variety of people to get an overview of this topic: (1) 60 interviews were conducted with gynaecologists, (2) we performed four focus group discussions with eight to ten Lady Health Workers providing maternal healthcare, and (3) we talked with family members of mothers who had died.

The study shows that delays in seeking care are related to poor socioeconomic status, limited knowledge about maternal care, and low incomes of rural people. The low status of women and male domination keeps women less empowered. In addition, early marriages and lack of family planning due to cultural values, religion and traditions stopped young girls from getting maternal healthcare.

The number of new mothers who die is very worrying in Pakistan. One of the important tasks for reducing deaths among pregnant women is to address certain socio-cultural practices. It is very important to reduce poverty and improve decision-making power to make sure women can use their right to medical care.

**Supplementary Information:**

The online version contains supplementary material available at 10.1186/s12978-021-01151-6.

## Background

Reducing maternal mortality is among the key determinants of development strategies for countries all over the world [1]. Globally, every two minutes a pregnant woman dies, either due to complications during pregnancy or in childbirth [2]. Almost 300,000 women died due to pregnancy-related complications in 2017. The great majority of these deaths (94%) occurred in countries with low resources [3]. With a population of approximately 204.6 million people, Pakistan is the sixth most populous country in the world. In Pakistan, the maternal mortality rate was 140 per 100,000 live births in 2017 [4]. Although there have been significant improvements in the country’s healthcare system, Pakistan still faces many challenges in relation to its high population growth, infant and maternal mortality, and many infectious and non-infectious diseases [5]. In terms of development indicators, Pakistan has shown poor performance, specifically with reference to maternal healthcare [6]. Maternal healthcare-seeking behaviour in Pakistan, particularly in rural areas, is deeply influenced and constrained by a series of religious and cultural factors [7].

Many studies have documented the association between religious, social and cultural beliefs and the health risks faced by childbearing women [7, 8]. For example, some studies on Muslim women show that they usually opt for ‘faith-based’ healthcare services. These services consist, to a large extent, of traditional or spiritual healers without an academic background to support their business. In particular, families who are impoverished and have limited access to education are marginalized from accessing biomedical healthcare services. This in turn creates distrust in the healthcare system and strengthens the preference for traditional healers. For that reason, women disregard proper medical attention during their pregnancy, resulting in negative effects on their reproductive health [9]. Globally, the development of science and technology has helped to overcome these practices associated with pregnancy. However, in most developing countries, people with low socioeconomic status continue to follow certain beliefs and cultural rituals, despite there being no scientific evidence available in their favour [10]. It has been significantly revealed that the societal norms, values and culture of any country has a substantial effect on its maternal mortality rate [10, 11]. All this is deeply rooted in the role and status of women within the society [12]. Pakistan is a strongly patriarchal society and thus men largely dominate household decision-making and finances. Women, particularly those living in rural areas and urban slums, are treated as subordinates and have limited or no say in personal and family matters [13]. The disadvantaged status of women and the male domination of society have adverse impacts on women’s reproductive health [14].

Worldwide, maternal healthcare systems have used numerous different approaches to improve the understanding of the difficulties faced by women during pregnancy or in childbirth. One of the most noteworthy of these approaches is the Three Delays Model [15]. According to research conducted by Thaddeus and Maine in 1994, complications during pregnancy can be avoided if adequate and timely treatment is provided. However, if treatment is delayed and/or insufficient, the consequences can be serious. These delays are threefold: (1) delay in making the decision to seek care, (2) delay in reaching a healthcare facility, and (3) delay in receiving the required maternal healthcare. The first delay is about recognizing maternal complications at the earliest possible time and making efforts to seek appropriate medical care immediately [15]. This type of delay is estimated to contribute to 73% of maternal deaths [16]. A huge body of research is available on maternal mortality in developing countries. Nevertheless, there is a paucity of literature on socio-cultural factors leading to maternal mortality within the context of the Three Delays Model—specifically the first delay. Thus, this study aims to explore the socio-cultural factors leading to a delay in seeking maternal healthcare, that is, the first delay, in maternal healthcare utilization in South Punjab, Pakistan.

## Methods

### Study design

We used a qualitative method for this study and performed three types of data collection with different target groups: (1) 60 key informant interviews with gynaecologists, (2) four focus group discussions with Lady Health Workers (LHWs), and (3) ten case studies among family members of deceased mothers.

### Study site

The study was conducted across all four districts of the division of Dera Ghazi Khan (DGK), South Punjab, Pakistan. The reason for selecting this division is that DGK remained lowest in significant socioeconomic indicators among all divisions of Punjab in 2017–18 [3]. For instance, 56.9% of the population of DGK is living below the poverty line, which is the highest proportion of all the divisions of Punjab province. Furthermore, the literacy rate among young women aged 15–19 years is only 48.9%, which is the lowest in the province. Similarly, the maternal mortality indicators reveal a very alarming situation for women in the area. Only 36.3% of women receive four or more antenatal care visits and only 29.8% of women have deliveries overseen by skilled birth attendants. Many women (43.0%) prefer traditional birth attendants (TBAs) for delivery and consultation during or after pregnancy, which is again the highest proportion of any division in Punjab province [3].

### Data collection

The 60 *key informant interviews with gynaecologists* at the principal hospitals in all four districts of DGK were performed by the first author. The interview guide (Additional file 1) was developed on the basis of a literature review and informal discussions with physicians. This interview guide consisted of questions concerning the socio-cultural factors contributing to a delay in seeking maternal healthcare—and in many cases eventually even leading to maternal death. The interviews lasted between 60 and 90 min. Interviews were conducted until we reached saturation point, where almost all responses were similar to those in previous interviews and no new information was emerging from the data. The interviews with healthcare professionals were conducted at their workplaces in the respective hospitals at a time of their choosing.

Considering the significance of LHWs, who have direct and close interactions with families and women in rural areas, four *focus group discussions with LHWs* were also conducted, one in each district. The LHW programme is a key part of Pakistan’s national strategy to reduce poverty and improve the health sector [17]. LHWs bring health services to the doorsteps of under-served and marginalized sections of society [18]. Therefore, LHWs are the community health workers who are most likely to provide healthcare information, including awareness of maternal health issues [19]. The list of LHWs within all four districts was obtained from the health department and contacts were made. The LHWs were requested to attend the principal hospital of the relevant district. They were facilitated and given a transport allowance by the research team. Eight to ten LHWs participated in each focus group discussion, which lasted between 60 and 90 min each.

In order to obtain the points of view of families where a maternal death had taken place, ten *case studies among family members of deceased mothers* were also conducted. The participants in these case studies were selected with the help of LHWs. They identified a person who had remained close to the deceased mother throughout her pregnancy and delivery. The interviews took place in the homes of the deceased mothers after receiving consent from the study participants. The maternal death case was included if the death had taken place during the previous two years in order to minimize recall bias. Maternal death was considered only if the death had been reported as an outcome of complications during pregnancy, in childbirth or during the postpartum period. To ensure valid responses, the interviewee had to be someone who had been close to the mother and was aware of all the details regarding the death. In most cases, the husband was reported as the main family member who had remained with the deceased mother. In a few cases, the mother-in-law or sister-in-law of the deceased mother were identified by LHWs as the main person who had remained with the deceased mother throughout her pregnancy and childbirth.

### Data analysis

All the interviews and focus group discussions were audio recorded and notes were taken during data collection. As interviews progressed, some participants used their native language of Punjabi (the regional language of Punjab province) and others spoke in Urdu (Pakistan’s national language). All the interviews were transcribed in Urdu and afterwards translated into English. Data analysis was performed manually using thematic analysis. All initial codes related to the research questions were joined together and transferred into a theme. The study used both a deductive and inductive approach for data analysis. During the analysis, a few themes were assessed based on previous literature related to the first delay in seeking care within the Three Delays Model developed by Thaddeus and Maine (deductive) [15]. As we continued the analysis process, inductive themes emerged from the data. Analytical induction and constant comparison of the categories were applied. Sub-themes and categories were themes that primarily emerged during notetaking, transcribing, translating and interpreting the data [20].

## Results

Table 1 presents the socio-demographic profile of the LHWs who participated in the study. Almost three quarters of the participants were aged between 30 and 40 years. The majority (77.8%) was married and about half (42.9%) had four or more children. Most of the LHWs (58.3%) were earning between 50,000 and 75,000 Pakistani rupees. One third (33.3%) had work experience of between five and ten years and a further 44% had work experiences of more than 15 years.

**Table 1:** Sample characteristics of Lady Health Workers (n = 36)

Variable	n	%
Age (in years)		
30–35	14	38.9
36–40	12	33.3
> 40	10	27.8
Marital status		
Married	28	77.8
Unmarried	8	22.2
Number of children (n = 28)		
0–2	6	21.4
3–4	10	35.7
> 4	12	42.9
Monthly income (in PKR)		
50,000–75,000	21	58.3
76,000–100,000	8	22.2
> 100,000	7	19.5
Education		
Matriculation	16	44.4
Intermediate	8	22.2
Bachelors	6	16.7
Masters and above	6	16.7
Work experience (in years)		
5–10	12	33.3
11–15	8	22.2
More than 15	16	44.4

1 US-Dollar = 162

*PKR* Pakistani Rupee

Table 2 presents the socio-demographic profile of the physicians—all of them women—working on gynaecology and obstetrics wards within the study area. The largest group (46.6%) of physicians was aged 30–35 years and 31.6% were aged 36 years or above. A great majority (70.0%) had an MBBS with specialization in gynaecology. About half of the sample (53.3%) had experience of working on gynaecology wards for 10 to 15 years. A significant proportion (70.0%) of the physicians was married. Among the married physicians, 66.6% had between one and three children. Additionally, 43.3% reported a monthly income between 100,000 and 125,000 Pakistani rupees per month.

**Table 2:** Sample characteristics of physicians working in gynaecology and obstetrics ward (n = 60)

Variable	n	%
Age (in years)		
30–35	28	46.7
36–40	19	31.7
> 40	13	21.7
Gender		
Male	0	0
Female	60	100
Marital status		
Married	42	70.0
Unmarried	18	30.0
Number of children (n = 42)		
0–2	28	66.7
3–4	11	26.9
> 4	3	7.4
Monthly income (in PKR)		
100,000–125,000	26	43.3
126,000–140,000	18	30.0
> 140,000	16	26.7
Education		
MBBS	18	30.0
MBBS and specialization in gynaecology	42	70.0
Work experience in gynaecology/obstetric ward (in years)		
5–10	1	1.7
11–15	32	53.3
> 15	27	45.0
Work station		
DHQ hospital	35	58.3
THQ hospital	25	41.7

1 US-Dollar = 162

*PKR* Pakistani Rupee

We investigated the social and cultural factors contributing towards delayed decision-making in seeking healthcare for women during pregnancy. The themes derived from the data analysis are presented below.

### Low status of women

Despite many governmental efforts at gender mainstreaming, Pakistan is far behind most nations in achieving gender equality in health, education, and economic and political participation for women. Women are still subjected to different forms of discrimination and have little or no say on personal or family matters [21]. The present study found that illiterate and socially isolated women were more vulnerable to poor reproductive and general health. There were certain social and cultural practices, such as *purdah* (the veil system), dependency on a male guardian, and other social restrictions on the independent mobility of women that deprived them of the ability to seek timely medical care during pregnancy and childbirth. A majority of LHWs (32 of 36) were of the view that, in rural areas, many women could not read or write because education was not considered necessary for them. One mother-in-law had a clear stance against women’s education, saying:

*Why bother with school or college education for girls? The ability to read and write is good but the most important thing is that they should be able to read the Holy Quran. And she should have the skills of cooking and home making. This is what every Muslim woman must learn.*

While discussing the healthcare needs of women, especially during pregnancy, the wife of a retired military spy said:

*During pregnancy, the family—especially the husband and mother-in-law—must be careful and considerate. If there’s a problem, it’s the duty of the husband to arrange a visit to a qualified care provider.*

### Lack of autonomy and mobility

In the local culture of Pakistan, women generally lack autonomy to seek care when they need it. It was noted that there were many “stakeholders” whose consent was necessary before a pregnant woman could embark upon seeking care from a health facility. One LHW explained:

*It’s not the decision of the pregnant woman when, why and from whom to seek healthcare. Usually, it’s the joint decision of many players, including the mother-in-law, father-in-law, husband and sometimes the husband’s brother. They make the decision according to the perceived severity of the illness, the cost, the nature of the threat, the availability and competence of care providers, and other conditions.*

A majority of physicians (50 of 60) reported that sometimes the decision to travel to a healthcare facility was not based on women’s health condition but on the availability of transport as well as the availability and willingness of their husband, father or brother to travel with them. A gynaecologist pointed out the restrictions on the mobility of women:

*Most of the time, mothers with pregnancy complications are brought to us [referring to gynaecologists] only when they’re near to death. Yesterday, I received a pregnant mother for delivery. She was in a critical condition with profuse bleeding. When I asked the family about this delay, they told me that they were waiting for her husband to bring her to hospital because culturally it’s forbidden for a woman to go alone.*

Another dimension of the lack of decision-making power is illustrated by one of the LHWs:

*Sometimes, women don’t want to make their decisions independently because it has serious consequences if something goes wrong. For example, if a woman selects a particular doctor for treatment and if the pregnancy is terminated by this treatment, the woman will be in trouble. So, she needs to take other family members into her confidence while making the decision.*

### Low nutritional status

Due to the low social status of women, they also experience discrimination at home. Sometimes, they are not provided with the proper food which is required during pregnancy, and their dietary needs are frequently ignored. A healthy and balanced diet during pregnancy and after childbirth is one of the significant factors affecting women’s health. The absence of good nutrition can have adverse effects on a mother’s health and can lead to maternal complications [22]. A gynaecologist in her early 30 s expressed her view on women’s poor nutritional status:

*I become horrified to see the pale ghostly faces of women who come to us with maternal complications. Giving food to the men within the house first, and then the children, is the cultural thing here. Women’s dietary needs are not their priority.*

The LHWs, being close to rural households, had their own observations to make about the obstacles causing the first delay in seeking healthcare for pregnant women. Some of the LHWs (15 of 36) were of the view that one of the main obstacles was poverty and powerlessness within the family power structure. A senior LHW said:

*In poor families, pregnant women can’t even get two decent meals; not to mention timely and proper medical care.*

Another middle-aged woman, who had retired from being an office attendant at the local middle school, said:

*Here, the main issue is not poverty but priority and preference. Women from a poor background—whose parents are poor and not influential—are not properly cared for in their in-laws’ homes. They are simply ignored; the issues of their health and illness are taken for granted.*

While probing families about any special focus on the pregnant mother’s diet, one mother-in-law commented:

*I can’t feed the pregnant mother first, if the children are crying out of hunger. My mother-in-law didn’t pay special attention to me nor did I with my daughter-in-law. What’s so special about giving birth?*

### Early marriages

In Pakistan, especially in poor rural families, early marriage, forced marriage and cousin marriage are common and considered normative cultural practices. A growing body of literature demonstrates the negative physical consequences of early marriages on young girls [23]. A few of the physicians (15 of 60) reported that child marriage was one of the main reasons for maternal mortality. According to them, a girl married at a young age was not mature enough to decide about her own healthcare and she was more dependent on her in-laws and husband for her healthcare needs. In the local culture, child marriage is justified by providing many reasons. One of the mothers-in-law expressed her strong belief in girls’ early marriage:

*Poor and powerless people are not safe here—so is the case with their daughters. We can’t afford to keep daughters unmarried for long at home.*

One of the LHWs said:

*This is a general perception here: The younger the girl is, the brighter are the chances of producing more children. Therefore, many people think that it’s a cultural thing, and they follow it.*

A female physician shared her views in this regard:

*In this area, girls are married at an early age and they have multiple pregnancies before reaching the age of 25 years. Sometimes I refuse to believe the age of the pregnant women when I’m told it. The fact is, they usually look 10 or 20 years older than their actual age. They’ve been producing children every year and the chances of maternal mortality with such health conditions are always higher.*

One LHW who had been working in a village community for the past ten years added:

*The poor parents are always in a hurry to marry off their daughters to lessen their burden. Culturally, people think marrying daughters early prevents them from becoming characterless. If girls remain unmarried after attaining puberty, there is a risk of creating affairs or sex scandals.*

One female physician emphasized the dependency which goes along with early marriage:

*I’ve assisted in the deliveries of many young mothers in this community. Frequently, they’re brought to us with pregnancy complications when it’s difficult to save their lives. They [referring to the young mothers] are not prepared to decide for themselves, about their family planning. And they don’t know anything about their reproductive health. They’re totally dependent on their in-law families for such decision-making.*

### Lack of reproductive autonomy

Due to their social exclusion and economic non-participation, women are not fully aware of their reproductive rights. In some areas, the birth of a baby girl is not welcomed; rather, it disempowers the mother who gave birth to a daughter. Therefore, a woman who is pregnant with a baby girl is less likely to seek appropriate and timely care during pregnancy. The present study showed a lack of family planning among married couples and that the average family size in rural households is large. A few of the case study participants (4 of 10) shared their beliefs that family planning is considered a sin in their religion. The majority of LHWs (30 of 36) were of the opinion that the desire for a son was the primary factor in a large family size. One husband with very poor socioeconomic status admitted:

*My wife died during the birth of our seventh child and I admit I never followed any family planning. I know very well it’s a conspiracy against Muslims.*

One LHW shared her experience and added:

*I was once physically abused by a mother-in-law and husband when a young married girl – who died in childbirth later – asked for a contraceptive pill, but tried to hide it from her family. Here, culturally and religiously, people think it’s a sin to follow family planning methods.*

Within the household power structure, mothers-in-law have more power. They can influence decisions related to the reproductive life of their daughters-in-law, including their health-seeking behaviour during pregnancy. When reproductive decisions are made by someone else and not by the mother, timely decisions about healthcare are difficult to achieve. The same observation was made by many participants (76 of 96) in terms of the role of mothers-in-law in the lives of their daughters-in-law. A female physician on a gynaecology ward revealed:

*The mothers-in-law are the ones who decide the next course of action once a pregnant mother is brought to us. I’ve even seen them insisting on saving the life of a baby in place of its mother, specifically if it’s a baby boy.*

When commenting on the situation, one LHW noted:

*When things are decided by the mother-in-law regarding seeking care, she has her own ‘agenda’. She may delay the visit to save money, to avoid travel or simply to settle a score with the pregnant mother. It’s unfair and doesn’t make sense; but this is how it is.*

### Poor understanding of pregnancy complications and risk factors

The present study found that women and their families were not very aware of pregnancy complications or the related risk factors. It is evident that a timely diagnosis of complications during pregnancy is possible, if antenatal visits are available for pregnant mothers [24]. However, the local culture has its own understanding of pregnancy and its associated processes. More than half of the LHWs (20 of 36) reported that many pregnant women had no opportunity to visit medical facilities for antenatal care in their local areas. Many of the LHWs (26 of 36) and gynaecologists (52 of 60) blamed the families for this situation. It was also noted that there is a lack of trust in certain diagnostic medical procedures performed at healthcare facilities for pregnant women. One female physician observed the following:

*Sometimes, there’s a serious lack of trust between doctor and patient. Some mothers are suspicious of ultrasound and think it’s a family planning device. Lack of trust is also a factor in delays to seeking formal care.*

While explaining the condition of rural women, one LHW stated:

*Poor women—who are the majority in this village—have no concept of prenatal care. They’re taken to healthcare facilities when something visibly serious happens to them such as bleeding, fits or they simply lose consciousness. For minor ailments, they’re treated at home.*

While describing the need to seek healthcare during pregnancy, one mother-in-law stated:

*Problems in pregnancy are normal and natural. Why rush to doctors for a natural process? For thousands of years, women have been delivering children at home. Doctors just complicate things to make money.*

One gynaecologist reported:

*Who cares about their treatment or antenatal check-ups? In the local culture, pregnancy is kept secret. A web of superstitions regulates the lives of pregnant women. They only come to us at a very critical stage.*

One of the sisters-in-law of a deceased mother revealed:

*My mother-in-law believes in keeping pregnant women inside the home. Therefore, she couldn’t be exposed to sunlight during the first three months of pregnancy. She did the same with my sister-in-law, who died in the fifth month of pregnancy due to some complication, because she was not allowed to go to a doctor due to her [referring to her mother-in-law] superstitious beliefs.*

### Seeking care within a pluralistic medical system

Like other developing countries, Pakistan has a complex pluralistic medical system in which the biomedical system coexists and competes with a host of indigenous medical systems, such as traditional healers (*hakeems*, folk healers, or spiritual healers). Depending on a patient’s social class, level of education, income and the perceived nature of their aliment, the patient selects a particular care provider, or multiple care providers, at any given time. A majority of the physicians (50 of 60) reported that getting multiple sets of advice and treatment from multiple care providers could cause a delay in seeking treatment from a qualified care provider. One physician noted:

*Women come to us with long-term complications such as high blood pressure or gestational diabetes. Poor women fail to understand the long-term treatment and ask for a quick remedy. Here come the traditional and spiritual healers: They promise quick relief.*

South Asian communities still attribute many physical and mental illnesses to the presence of supernatural powers and seldom hesitate to consult spiritual healers [8]. The role of spiritual religious leaders, even in providing consultations for medical care during pregnancy, is common. One of the respondents, who was a sister-in-law of a deceased mother, and who was also pregnant said:

*I’ve been advised by peer sahib [referring to spiritual healers] to keep a knife under my pillow and avoid sunlight throughout my pregnancy. My mother-in-law believes that it could save me from future complications.*

While explaining the mechanism of decisions being delayed by spiritual healers, one LHW added:

*I was once called upon to see a woman who had profuse bleeding in her fifth month of pregnancy. While probing, I learned that she was stopped by a local peer [referring to a spiritual healer] from travelling outside her home or consulting a doctor as she ran the risk of being attacked by evil forces. She died the same evening.*

The mother of a deceased women shared:

*Here, we have a strong belief in nazar lagna [the evil eye]—especially during pregnancy. My daughter’s mother-in-law did not allow her to go outside the home or consult a doctor. Always the Dai [a TBA] came to her home to provide treatment. But she [referring to the Dai] did a very bad job with my daughter. She took my daughter’s life. She and my daughter’s in-law’s family are responsible for her death.*

A physician on a gynaecology ward expressed great disappointment in the large adverse impact of cultural and religious beliefs on pregnant women:

*These spiritual healers are part of the religious and cultural beliefs of rural people. Sometimes, a husband comes or not. But when a pregnant mother is close to death and is brought to us, the local traditional healer accompanies the family and even intervenes in our treatment methods.*

Spiritual healers of various types influence the health belief systems of women during pregnancy, which in turn regulates their care-seeking behaviours [25]. Frequently, pregnant women and their families in villages are dependent on TBAs for healthcare. The families in our study greatly preferred TBAs and sought help from them for women during pregnancy. A mother-in-law in her late 70 s showed her great trust in these TBAs:

*Let us not break with tradition. In the four walls of our house we get great help from Dai [referring to TBAs] during pregnancy and for delivery. The poor Dai is happy to receive a few kilos of Atta [wheat flour] as her fee, even after delivering the baby. I’m happy they’re always available for us.*

An experienced LHW, however, expressed her anger about the role of TBAs:

*I’m helpless when I see these Dais treating cases of preeclampsia and eclampsia [referring to high blood pressure during pregnancy] with herbal medicine. They don’t hesitate to cut the baby’s umbilical cord with a knife used for vegetables. Families blindly trust in them and no force can stop them.*

The gynaecologists shared their own experiences of dealing with complicated cases brought to them after treatment from untrained TBAs. One senior gynaecologist shared her views by commenting:

*The half-dead pregnant mothers are sometimes brought to us with serious complications. Most of the time, they are brought to us after inappropriate interventions by untrained Dais, and Dais are unable to handle the delivery. In my eyes it’s a killing, it’s a murder.*

## Discussion

It is a globally accepted phenomenon that the factors determining health behaviours are found in different contexts. These can be physical, social, economic or cultural in nature [26]. The same goes for maternal healthcare in Pakistan, particularly in rural areas. This study found that various socio-cultural factors contribute towards the first delay in decision-making (delay in seeking care) about appropriate maternal care, which has an impact on maternal mortality in South Punjab [15]. Many studies have found that the first delay is the most significant contributor to maternal deaths [16, 27]. The qualitative data gathered in this study shows that healthcare-seeking behaviours during pregnancy are extremely complex and embedded in a cultural and indigenous belief system. Additionally, prevailing economic conditions, patriarchal ideology, and the role and status of women within the family power structure also influence women’s healthcare-related decision-making [28].

We argue that the low socioeconomic status of women is one of the major obstacles affecting women’s decision-making in seeking healthcare during pregnancy and childbirth in rural areas and urban slums [28]. This low social status of women exposes them to multiple negative social and cultural practices, such as early marriage, multiple and closely spaced pregnancies and domestic violence [29]. We observed that women’s strong socioeconomic dependency on men throughout their lives hinders them from deciding about their own health and wellbeing. The limited autonomy of women, their lack of education, and their dependency on men leaves them at the mercy of their husband’s family to look after them during or following pregnancy [30]. We found that women were not provided with sufficient nutrition during pregnancy. This indicates that poverty or lack of resources is a prime factor affecting women’s health. A healthy and balanced diet during pregnancy and after childbirth is one of the most important factors for women’s health. The absence of an adequate and balanced diet can have adverse effects on a mother’s health and can lead to maternal complications [22].

Cultural restrictions on women’s mobility are another barrier noted in our study. Restrictions on women’s mobility during obstetric emergencies may lead to a delay in seeking timely care [31]. The women were found to observe *purdah* (the veil) under all circumstances and were not allowed to travel outside the home without men even during a health emergency. The consent of “stakeholders”—i.e., the husband’s and mother-in-law’s permission—was revealed as necessary for a woman in relation to significant events in her life—ranging from family planning issues to her choice about consulting an appropriate maternal caregiver [32]. Women going out alone due to a medical emergency are thought to bring great dishonour to the family. Making a timely decision about selecting the right kind of healthcare provider is critical for avoiding maternal and child deaths. Unfortunately, for some poor rural women, this decision is not simple [33]. Our study highlights that delays in seeking care for obstetric complications are influenced by traditional ‘wait and see’ tactics. The action of seeking medical care is usually undertaken only when the situation is already out of control or the condition of the pregnant woman worsens [27].

It was also noted that the practice of deciding family size was also in the hands of the mother-in-law or the husband, for cultural reasons. Many studies have highlighted the influential role of mothers-in-law and husbands in women’s lives regarding decisions about family planning and giving birth to children [33, 34]. Among others, the lack of family planning among rural couples was found to be a pivotal factor resulting in high maternal deaths [33]. The participants in the case studies reported multiple socio-cultural factors behind the low use of family planning methods and short birth spacing amongst married couples. The religious interpretation regarding family planning among rural couples was an important element, as has already been explained in previous research [34]. Rural people believe that the use of family planning methods and restricting family size is against the preaching of Islam. A few reported the use of family planning as a conspiracy against Muslims to limit their population growth [35]. A large majority of religious groups in developing countries do not favour birth control methods and call them un-Islamic and unnatural [36]. Another cultural factor noted for leading to a large family size was families’ desire for a son [37]. The present data reveals that this particular desire pushed women to give birth almost every year. Multiple pregnancies in turn lead to higher risk.

This study also shows that Pakistan, with its patriarchal society, has visibly delineated roles related to gender. The household structures in villages, where mothers-in-law and husbands hold major positions, hardly allow women any power to make decisions regarding their own lives [38]. A similar influence was found in relation to the use of antenatal care visits. Our results indicate very low use of antenatal care, which greatly contributes to the first delay. A growing body of knowledge has revealed a lack of awareness amongst women and their families about the delivery and continuity of antenatal care [39]. Some of the results that have emerged from this study, including the low use of antenatal care, are similar to studies conducted in other South East Asian and South Asian countries [40–43]. The data from this study also indicates the strong cultural trait of early marriage. Early marriages were seen to be the root cause of multiple pregnancies. These are the notable social and cultural conditions that increase the lifetime risk of maternal death [44]. The primary cause noted for early marriage in rural areas was the perception of daughters as a burden. There is suspicion about the character of girls if they remain unmarried for too long. The fact that girls were young and under the influence of their mothers-in-law and husbands was found to be an important factor in their inability to seek the desired maternal care [44]. Previous studies have shown that bearing a child at an early age increases the risk of several medical complications and, therefore, leads to an increased risk of complications during pregnancy or immediately after childbirth [44, 45].

In addition to that, the culture of relying on TBAs is a common social and cultural practice in rural areas of Pakistan [46]. Dependency on TBAs is found to be a great barrier to seeking appropriate maternal care. The majority of TBAs are largely untrained and without clinical expertise to handle complicated situations during pregnancy or childbirth [46]. This study documents that rural communities are inclined towards their centuries-old traditions of trusting and utilizing the services of TBAs. They are the preferred choice, and families are not ready to compromise on that. Families find them easier to afford and it is convenient to call them when needed. In addition, they are available 24 h a day and provide services on the doorstep [47]. The issue of *purdah* (the veil) is also not compromised in such cases, because it saves women from going out to consult physicians, who might in some cases be male. However, a previous study attributed complications during pregnancy or after childbirth to uneducated and untrained TBAs [48].

This study has also shed light on traditional and customary beliefs about spiritual healers. From medication to advice on the daily matters of life, the advice of spiritual healers is considered highly important. As a matter of fact, this is known to be a social issue in rural areas [49]. Visiting these spiritual healers in rural communities is another ritualistic practice in rural areas that prevents women from seeking maternal care from medical practitioners. In addition, a strong belief in witchcraft or the evil eye directed against pregnant women is a major component of many cultures [50–53]. In order to ward off evil spirits and the influence of supernatural powers on pregnant women, the elderly of the family, most frequently the mothers-in-law, take the decision to consult spiritual healers [54, 55]. Rural people believe in particular rituals and practices, such as the chanting of certain verses, a spiritual healer’s blow to the face or body of a woman, or simple water as a great medication for maternal complications [55]. These spiritual healers were found to be influential figures in rural women’s lives in decisions ranging from the place of birth to permission for her mobility during pregnancy and family planning. This practice is very common in Pakistan and families prefer spiritual healers over professional and trained physicians [55, 56]. Therefore, it might be advisable to include TBAs more in the medical health system by providing them with adequate training. Since TBAs have access to a large part of the population, they may be able to transfer knowledge about the impact of appropriate maternal healthcare. This may change sociocultural beliefs in the long term.

### Limitations

One of the major strengths of our study is the large sample size for a qualitative study and the use of three different types of data collection (key informant interviews, focus group discussions, and case studies) that included diverse groups of interviewees. The synthesis of these results allows for an in-depth examination of the social and cultural factors associated with maternal mortality. However, one needs to keep in mind that these results describe the specific situation in a rural and impoverished region of Pakistan. A further limitation is associated with the data analysis. The thematic analysis is based on what was spoken during the interviews and focus groups. However, attitudes that were not expressed aloud and non-verbal information are not included in the data analysis.

## Conclusion

Maternal health in Pakistan is strongly influenced by socio-demographic elements, societal structures, cultural practices and religious beliefs, gender discrimination and the lack of autonomy among women. The situation of maternal mortality is very alarming in the country. The uphill task of reducing deaths among pregnant women is deeply embedded in addressing certain sociocultural practices which are constraints for women seeking maternal care. Despite governmental efforts to provide maternal care to rural women in Pakistan, social practices and cultural beliefs play important roles in deciding which women will survive and which will not. It is absolutely pivotal to identify the causes of maternal deaths as early as possible. Maternal deaths can be easily prevented if women are saved from putting off seeking care. The important key to reducing maternal mortality is to address the poor economic and social status of rural families. A strong emphasis is required on raising the status of women in their communities through education and economic empowerment. Without addressing the social and cultural practices in the broad integrated strategies aimed at improving maternal health in Pakistan, the average Pakistani mother will continue face a high risk of maternal mortality and will leave behind tales of misery, discrimination and vulnerability.

## Supplementary Information

**Additional file 1.** Interview guide.

## Data Availability

Pseudonymised transcripts are available from the corresponding author upon reasonable request.

## Abbreviations

DGK: Dera Ghazi Khan
LHW: Lady Health Worker
PKR: Pakistani Rupee
TBA: Traditional birth attendant
