# Supplementary material for: The influence of social and cultural practices on maternal mortality: a qualitative study from South Punjab, Pakistan
Source: Reprod Health. 2021 May 18;18:97. doi: 10.1186/s12978-021-01151-6 (PMC8130310; doi:10.1186/s12978-021-01151-6)
Supplement: Supplementary file 1 — Additional file 1. Interview guide. [file 12978_2021_1151_MOESM1_ESM.pdf]

Key informative interview guide for physicians

1. What are the socioeconomic characteristics of a rural household in Dear Ghazi Khan?
2. What is your opinion on maternal mortality cases in Dera Ghazi Khan?
3. What are the social factors that are hurdles for rural women to avail maternal health care services in the light of first delay of maternal mortality (seeking care)?
4. What are the specific religious and cultural patterns/practices that stop women to seek an adequate health services in the context of first delay of maternal mortality (seeking care)?
5. What is the general status of rural women in of Pakistan?
6. What are the reasons that prevent women and families to seek maternal care from hospitals?
7. Do you think your health facility is equipped to deal with maternal emergencies?
8. What do you say about display of certain behaviors of families who bring women with obstetric complications to you?
9. Do you have any problem working in backward areas?
10. From your opinion: What measures can be taken to reduce maternal mortality in Pakistan?

Focus group discussion guide for lady health workers

1. What do you know about the socio-economic demography of rural people of Dera Ghazi Khan?
2. What do you know about the occurrence of maternal mortality in the area?
3. What are the contributory social factors that are obstacle in maternal mortality in seeking (first delay) a right adequate maternal health services?
4. What are the contributory cultural factors that prevent women in seeking (first delay) a right maternal health care service?
5. What are your views specifically on the status of women? How do you see the status of women in the area?
6. What are the reasons that prevent women for seeking ante/post natal care?
7. What are the preferred choice of family for consultancy in obstetric complication and deliver?
8. What type of household structure exist in rural areas?
9. What are certain religious and cultural practices exist in rural area foe pregnant women?
10. What problems you face while working with rural community during your awareness campaigns?
11. What do you suggest to improve maternal health conditions and what measures can be taken to reduce maternal mortalities in rural Pakistan?

Interview guide for case studies

1. What is the socio-economic background of the family?
2. What was the socio-economic background of the deceased mother?
3. What is the average age when women of the area get married including the age of the deceased mother?
4. How many children did the deceased mother leave after death?
5. What have been the status of anti/post natal care in pregnancies?
6. How much family has focused on the overall health of deceased mother including her dietary patterns during pregnancies?
7. Has the deceased mother been the part of the decision making on significant issues including decisions on her reproductive health?
8. What have been the preferred choices of family to consult in case of emergency during pregnancy?
9. What are the preferred choices for delivery in rural areas and why?
10. What are certain cultural and religious traits/practices rural people think important to follow during pregnancy of women?
11. What important role does the mother-in-law or elderly women of the house play during pregnancies of the daughter-in-law?
12. What have been the hurdles in seeking right maternal care during pregnancies?
13. What was the major cause of death of the mother?
